# Outcomes of Transarterial Embolisation (TAE) vs. Transarterial Chemoembolisation (TACE) for Hepatocellular Carcinoma: A Systematic Review and Meta-Analysis

**DOI:** 10.3390/cancers15123166

**Published:** 2023-06-13

**Authors:** Alexander Lawson, Sivesh K. Kamarajah, Alessandro Parente, Kamil Pufal, Ramanivas Sundareyan, Timothy M. Pawlik, Yuk Ting Ma, Tahir Shah, Salil Kharkhanis, Bobby V. M. Dasari

**Affiliations:** 1 Birmingham Medical School, University of Birmingham, Birmingham B15 2TT, UK; awl639@student.bham.ac.uk (A.L.);; 2 Department of HPB and Liver Transplantation, Queen Elizabeth Hospital, Birmingham B15 2TH, UK; siveshkk93@gmail.com (S.K.K.);; 3 Department of Radiology, Queen Elizabeth Hospital, Birmingham B15 2TH, UK; 4 Department of Surgery, The Ohio State University, Wexner Medical Center, Columbus, OH 43210, USA; 5 Department of Oncology, Queen Elizabeth Hospital, Birmingham B15 2TH, UK; 6 Liver Unit, Queen Elizabeth Hospital, Birmingham B15 2TH, UK; 7 Institute of Immunology and Immunotherapy, University of Birmingham, Birmingham B15 2TT, UK

**Keywords:** hepatocellular carcinoma, transarterial embolisation, transarterial chemoembolisation

## Abstract

**Simple Summary:**

Non-surgical management of hepatocellular carcinoma (HCC) is used for selected patients. Of these management options, transarterial embolisation (TAE) and transarterial chemoembolisation (TACE) are the two main locoregional treatment options. There was no difference in OS among patients treated with TACE/TAE, single versus repeat treatments. Post-procedural adverse effects were higher in the TACE group but were not statistically significant. TACE has a comparable long-term survival and complications profile to TAE for patients with HCC. However, the low-to-moderate quality of current RCTs warrants high-quality RCTs, which are necessary to provide enough evidence to give a definitive answer and inform treatment plans for the future.

**Abstract:**

Although hepatocellular carcinoma is increasingly common, debate exists surrounding the management of patients with unresectable disease comparing transarterial embolisation (TAE) or transarterial chemoembolisation (TACE). This study aimed to compare the outcomes of patients receiving TAE and TACE. A systematic review was performed using PubMed, Medline, Embase, and Cochrane databases to identify randomised controlled trials (RCTs) until August 2021. The primary outcome was overall survival (OS) and the secondary outcomes were progression-free survival (PFS) and adverse events. Five studies with 609 patients were included in the analysis. There was no statistically significant difference in the OS (*p* = 0.36) and PFS (*p* = 0.81). There was no difference in OS among patients treated with a single TACE/TAE versus repeat treatments. Post-procedural adverse effects were higher in the TACE group but were not statistically significant. TACE has comparable long-term survival and complications profile to TAE for patients with HCC. However, the low-to-moderate quality of current RCTs warrants high-quality RCTs are necessary to provide enough evidence to give a definitive answer and inform treatment plans for the future.

## 1. Introduction

Hepatocellular carcinoma (HCC) is the sixth most common cancer worldwide, with a rising incidence and an increasing global health burden over the last decade [1,2]. For patients with HCC within Milan criteria, preferred curative options include either liver transplantation or resection, with a reported 5-year survival of 65–80% [3,4]. Liver transplantation is the curative option for hepatocellular carcinoma (HCC) for patients with background liver disease and 20-25% of the LT performed in Western countries are for HCC. The selection criteria of HCC candidates reminas fairly rigid despite of some extended criteria applied to the well accepted Milan criteria. Surgical resection is not often possible in the presence of background liver disease and portal hypertension and the criteria for liver transplantation are rigid in order to obtain best oncological outcomes. For patients who are not suitable for surgical treatment, the management options are usually based on the degree of background liver disease, and the extent of tumour load, including portal venous involvement.

Transarterial embolisation (TAE) and transarterial chemoembolisation (TACE) are the two main locoregional treatment options in the management of unresectable HCCs, to palliate or downstage or to bridge to transplantation. Liver embolization for HCC is commonly used in two main settings: (1) large unresectable HCCs unsuitable for surgery or ablation, and (2) prior to resection or to liver transplantation as a bridge therapy. The vascular supply to the HCC tissue is predominantly arterial and the selective blockade of arterial flow using superselective angiography is used as a strategy to cause necrosis of tumor tissue. 

TAE aims to reduce tumour burden through the embolisation of the arteries that predominantly feed the tumour. In TAE, superselective vascular embolization using gelatin sponge, Lipiodol, or microparticles as small as 40μm in diameter with no drugs are injected. TACE involves the additional administration of cytotoxic chemotherapy to the tumour cells in addition to embolisation. Principally, both these treatment modalities lead to hypoxia-induced necrosis of the tumour [5,6]. A variety of materials are used for embolization to slow tumor progression. During TACE procedure, tumor is filled with a chemotherapeutic drug such as doxorubicin, epirubicin, cisplatin, or mitomycin C along with Lipiodol or drug-eluting beads. In general, the best candidates for these procedures are those patients with unresectable lesions without vascular invasion or extrahepatic spread and with well-preserved liver function.

The relative potential superiority of TACE and TAE for patients with the unresectable disease remains unclear. In particular, data in favour of the superiority of TACE over TAE are still lacking [7]. A RCT comparing conventional TACE, TAE, and best supportive care (BSC) was prematurely terminated because of the superiority of TACE over BSC, which prevented the ability to determine the efficacy of TAE relative to TACE, which could only be hypothesised based on the trend observed in overall survival (OS) [8]. In addition, other RCTs published more recently on this topic seem to provide discordant results [9,10]. These discrepancies may be due to a number of unsolved issues concerning TACE. For example, the optimal chemotherapeutic agent to inject, as well as the efficacy of drug-eluting embolic agents, are still a matter of debate [11,12]. The objective of the current study was to perform a systematic review and meta-analysis comparing RCT data on TAE and TACE among patients with unresectable HCC.

## 2. Methods

### 2.1. Search Strategy

A systematic search of PubMed, Embase, and the Cochrane Library databases was conducted up to August 2021 by four independent investigators (SKK, AP, AL, and KP). The search terms included ‘transarterial embolisation’ or ‘transarterial chemoembolisation’ or ‘chemoembolisation’ and ‘survival’ and ‘hepatocellular carcinoma,’ individually or in combination (Table 1). The ‘related articles’ function was used to broaden the search, and all citations were considered for relevance. A manual search of reference lists in recent reviews and eligible studies was also undertaken. 

### 2.2. Inclusion and Exclusion Criteria

Inclusion criteria were (1) studies that compared TACE and TAE relative to overall survival in human subjects with HCC; (2) published in the English language; and (3) RCTs. Exclusion criteria were (1) conference abstracts, review articles, and case reports (<5 patients); (2) publications with mixed populations in which the outcomes of patients could not be separated by disease type (HCC) or patient population (i.e., resectable or unresectable); and (3) no reported survival data.

After excluding duplicates, four researchers (SKK, AP, AL, and KP) independently reviewed the titles and abstracts of studies identified by the literature search. Full copies of publications considered potentially relevant to the research question were obtained for further review. The reference lists of all included studies were hand-searched to identify other potentially relevant studies. Any areas of disagreement were resolved through discussion until consensus was achieved. 

### 2.3. Study Outcomes

The primary outcome measures were OS defined as the interval between TAE or TACE and death or censoring of data. Secondary outcome measures were progression-free survival and adverse events (i.e., cholecystitis, liver abscess, liver failure, and leukopenia).

### 2.4. Data Extraction

Four researchers (SKK, AP, AL, and KP) extracted data on study characteristics (author, year of publication, country of origin, and patient numbers), patient demographics (age, sex, and TACE/TAE details), and OS. Any disagreements were resolved through discussion with other authors until consensus was achieved (SKK, AP, and BVMD). Where data were missing for some of the outcomes in the included studies, analyses were performed with the reported numbers for those outcomes. 

### 2.5. Assessment of Methodological Quality 

The methodological quality and quality of reported outcomes were assessed by four independent researchers (SKK, AP, AL, and KP). Methodological quality was formally assessed using the Cochrane tool for publication bias for randomised studies (SKK, AP, AL, and KP) [13,14]. Any disagreements were resolved through discussion amongst authors until consensus was achieved (SKK, AP, and BVMD).

### 2.6. Statistical Analysis 

The systematic review and meta-analysis were conducted in accordance with the recommendations of the Cochrane Library and PRISMA guidelines [15]. For random effects, the DerSimonian–Laird method was used for the meta-analysis of RCTs. Reported Hazard Ratios (HR) were used directly in the quantitative meta-analysis. Standard errors for HRs were calculated from the 95% CI when provided in the article. Funnel plots were used to visually assess publication bias. Heterogeneity among studies was assessed using the I^2^ value to determine the degree of variation not attributable to chance alone. I^2^ values were considered to represent low, moderate, and high degrees of heterogeneity where values were <25%, 25–75%, and >75%, respectively. Funnel plot asymmetry was assessed using the Egger test. Statistical significance was considered when *p* < 0.05. Data analysis was undertaken using R Foundation Statistical software (R 3.2.1), as previously described [16].

## 3. Results

### Study Characteristics

**Figure 1:**
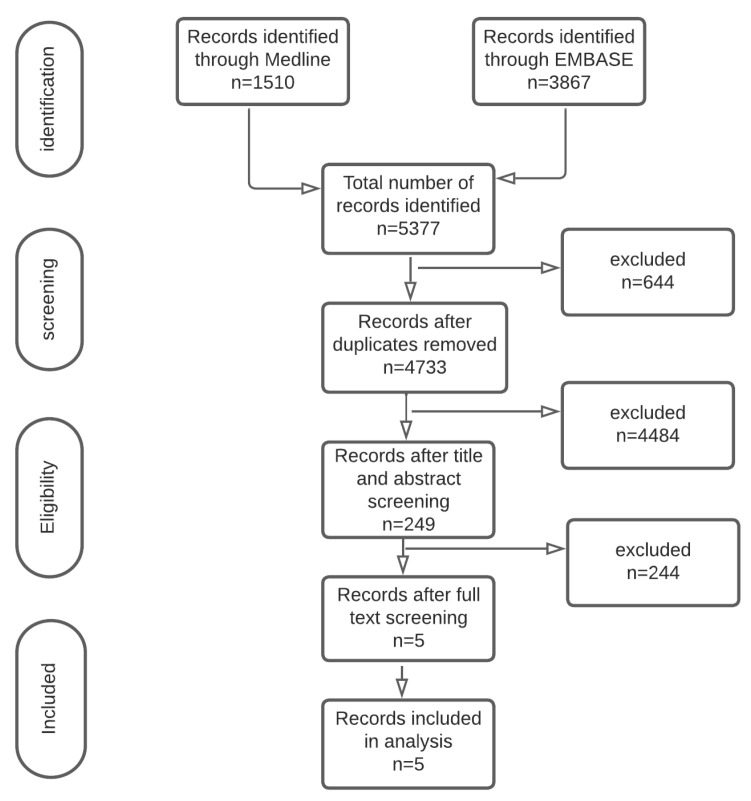
PRISMA diagram.

Among the 5377 studies identified, 229 studies had full text reviewed; five studies [9,10,17] with 609 patients were included that reported on patients undergoing TACE or TAE for unresectable HCC. A summary of included studies is presented using the PRISMA diagram (Figure 1). 

The baseline demographics of patients [9,10,17,18,19] are presented in Table 2. 

Quality assessment of the included studies according to the Cochrane Risk of Bias is presented in Table 3 of publications published from 1992 to 2016. Studies were completed in different countries with all studies being completed in secondary/tertiary care (or local equivalents). Studies were published in a variety of high-impact journals.

## 4. Survival Outcomes

### 4.1. Overall Survival 

**Figure 2:**
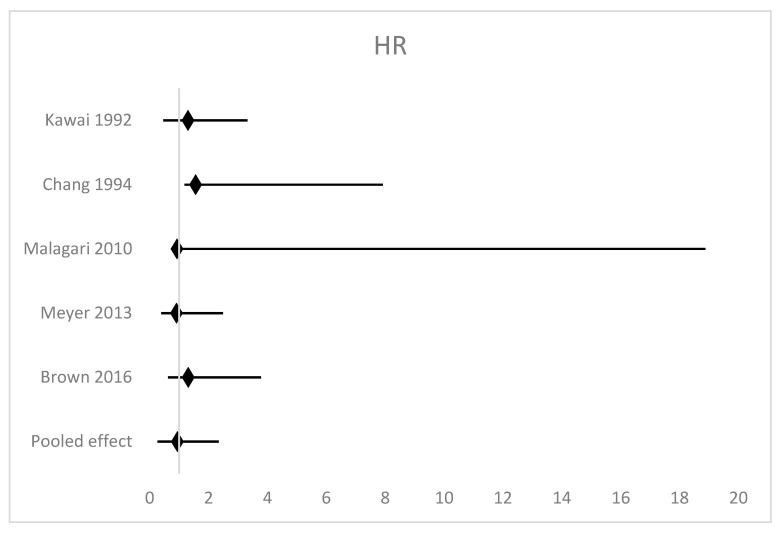
Forest plot of the meta-analysis of overall survival [9,10,17,18,19].

There were no data to support the superiority of either TACE or TAE among patients with HCC. Overall survival was reported in all five studies [9,10,17,18,19]. Hazard ratios ranged from 0.91 to 1.56 with no single study reporting a statistically significant difference in OS. A random effects model meta-analysis (Figure 2) demonstrated a non-significant pooled effect measure of HR 1.14 (95% confidence interval (CI) 0.87–1.46 *p* = 0.36). There was low heterogeneity in this analysis with I^2^ = 0%.

### 4.2. Comparison by Number of Sessions

Sub-group analysis was performed based on whether patients received a single intervention or multiple repeat interventions. A single TACE/TAE session was used in two studies (328 patients). In these studies, a non-significant HR 1.32 (95% CI 0.87–2.01 *p* = 0.19) was observed. Similarly, for patients who underwent repeat TACE/TAE (271 patients), TAE was associated with a non-significant HR 1.06 (95% CI 0.70–1.61 *p* = 0.77); there was no difference in overall survival among patients treated with a single TACE/TAE versus repeat treatments.

### 4.3. Progression-Free Survival

Progression-free survival was reported by two studies [9,17]. These studies carried similar weights within the analysis. HR for the two studies ranged from 0.87 to 1.36, although neither study demonstrated a statistically significant result. Hazard ratios were used to calculate the pooled effect which was statistically non-significant [HR = 1.05 (95% CI 0.68–1.62 *p* = 0.81)]. Moderate levels of heterogeneity were observed in this analysis (I^2^ = 29%). 

### 4.4. Comparison by Chemotherapeutic Agent

Subgroup analysis by chemotherapeutic agent used (Table 1) in TACE was performed. Doxorubicin was the chemotherapeutic agent in two [10,17] of the included studies. The pooled effect measure of these produced a result more in favour of TAE; however, it was not statistically significant, with HR 1.30 (95% CI 0.91–1.86 *p* = 0.16). This result also had no heterogeneity (I^2^ = 0%). For studies [9,18] using Cisplatin as the chemotherapeutic agent (228 patients), the pooled effect favoured TACE with HR 0.97 (95% CI 0.67–1.41 *p* = 0.87). Again, this result was not statistically significant.

### 4.5. Adverse Events

Ten common adverse events were included in this review. A summary of the results of the meta-analyses is detailed in Table 4. The severity of grading of complications was only presented by two studies [9,17] using different grading measures. The analysis was therefore performed without differentiating the grades of adverse events.

### 4.6. Cholecystitis

Cholecystitis was reported in three studies [10,17,18] including 231 patients. The Peto odds ratios ranged from 0.14 to 7.95. The pooled effect of this analysis was a non-significant Peto odds ratio = 0.70 (95% CI 0.12–4.06) associated with high levels of heterogeneity (I^2^ = 59%).

### 4.7. Liver Abscess

The incidence of the liver abscess was reported in three studies [9,10,17] including 271 patients. No study reported a statistically significant result. Two studies favoured TACE, whereas one favoured TAE. All studies had high degrees of uncertainty with large confidence intervals. Peto odds ratios ranged from 0.13 to 2.08 with a non-significant pooled effect of 0.51 (95% CI 0.10–2.58 *p* = 0.42) and moderate heterogeneity was observed (I^2^ = 30%).

### 4.8. Liver Failure

The incidence of liver failure was reported in three studies [9,10,17] (271 patients). Two studies favoured TAE and one study favoured TACE, with no significant difference in the overall analysis. There was a notable degree of uncertainty in the results from the studies with a wide 95% CI calculated. The Peto odds ratio ranged from 0.14 to 1.89 and the pooled effect of these studies is 1.01 (95% CI 0.25–4.11 *p* = 0.99). No heterogeneity was observed (I^2^ = 0%) at two degrees of freedom (Chi^2^ = 1.28)

### 4.9. Abdominal Pain

Incidence of abdominal pain was reported in two studies [9,19] (335 patients). Both studies favoured TAE with the Peto odds ratio ranging from 1.03 to 1.58. Both results were not statistically significant. The meta-analysis gave a pooled effect Peto odds ratio of 1.12 (95% CI 0.71–1.78 *p* = 0.62). No heterogeneity was observed in this study (I^2^ = 0%) with one degree of freedom (Chi^2^ = 0.56)

### 4.10. Fever

Incidence of fever is reported by two studies [9,19] (335 patients). One study favours TACE, whereas the other favours TAE. Peto odds ratios for this outcome range from 0.29 to 1.04. The pooled effect of this meta-analysis gave a non-significant Peto odds ratio of 0.68 (95% CI 0.41–1.13 *p* = 0.13). Very high levels of heterogeneity were observed (I^2^ = 81%) with one degree of freedom (Chi^2^ = 5.29)

## 5. Discussion

Although TACE is considered the standard of care for BCLC intermediate-stage HCC patients, robust data in favour of a clear superiority of TACE (with chemotherapy) over TAE (bland embolisation) are still lacking. The well-known hypervascularisation of HCC nodules provides the rationale for the occlusion by embolic particles, which results in tumour hypoxia and necrosis. However, whether the addition of local chemotherapy has an additive anti-tumour effect is still a matter of debate [20,21,22]. The current study demonstrated TAE to have a comparable survival and morbidity profile compared with TACE. 

Side effects of liver embolization include pain, nausea, fatigue, fever, and transient elevations of asparate aminotransferase, alanine aminotransferase, and bilirubin levels. Symptoms are usually self-limiting. There was, however, a decreased odds of emesis in the TAE group and increased odds of experiencing anaemia in the TACE group, which are expected adverse effects of chemotherapy. Serious complications such as hepatic failure, gastroduodenal ulceration, kidney failure, and death (2–3%) have been reported. 

Trials comparing TAE versus TACE included different size cohorts, patient populations, and tumour stages. For instance, some studies included patients with vascular involvement by the tumour, where the benefit of TACE or TAE is debatable and the survival can be influenced by the extent of tumour irrespective of the endovascular embolising comparators that are evaluated [23]. The embolising agent varied over time in recent decades, hence the different sizes and the different embolising techniques may have led to a lack of demonstration of efficacy due to dilution bias and high heterogeneity in the meta-analysis of data [23,24]. This is particularly relevant considering that the time range in which the included studies were retrieved spanned from 1988–1989 to 2007–2012 for the rest of the study period to 2021. Including the studies over this period would have accounted for the variability. It will be interesting to know the outcomes of the ongoing trials that assess the role of combined immunotherapy and TACE. Technical aspects of the procedures have evolved with more selective embolisation in recent times, as is the selection of patients for repeat procedures to those only with residual tumour activity. Hence, the strength of the conclusion from this study is limited by such intrinsic shortcomings. At the same time, a more restricted selection of the literature would lead to a too-limited amount of data to be analysed. Hence, at present, there is no better way to provide data on the comparison of survival outcomes between TACE and TAE and some of these results need further validation in future studies. 

Understanding tumour response to both TAE and TACE will allow a better understanding of patient selection for either treatment arm. However, the studies included in the present review lack a standardised approach to reporting this, precluding a reliable assessment. The well-established tools to assess tumour response are the RECIST and mRECIST systems. Many of the included studies pre-date the use of RECIST tool and hence a variety of regional or radiological cut-offs to define patient response to treatment. With the global challenge of increasing HCC incidence, the need for internationally translatable research is necessary, and hence more randomised studies using consistent measures. Furthermore, identifying non-responders helps choose alternative treatment options. 

It will need further research regarding the ability to predict the candidates who would respond to the embolization procedures and who could have complete tumor ne crosis on the initial or repeat procedures. The prognostic indices that are currently in use are based on clinical and laboratory parameters. These are useful in assessing the safety of the procedure but there is a need for further discrimination based on the tumor morphological characteristics and molecular markers to assess response to TACE or TAE. Early work demonstrated that transcriptome profiling to identify unique gene signatures of naïve HCC cells can be a predictor of response to treatment [25]. The adaptive mech anisms resulting from embolization procedure induced hypoxia have been investigated – hypoxia-inducible factor 1-α (HIF1α) and vascular endothelial growth factor (VEGF) for neoangiogenesis, CD34 for microvessel density, CA9 for antiapoptotic activity, CD133 and nestin for stem cell features, and vimentin and E-cadherin for the epithelial–mesenchymal transition. However, on multivariate analysis it is found that only CD34 and VEGF retained a significant association with TACE response. A typical pattern of expression (VEGF–, CD34+) was associated with resistance to TACE, suggesting that HCCs with this expression pattern are more resistant to hypoxia because they have developed a complete vascular network (increased CD34) without requiring further neoangiogenesis (decreased VEGF). These include HIF 1-alpha, which is upregulated in non-responders and contributes to neovascularisation, making TACE or TAE ineffective. 

In the East where Hepatitis B virus (HBV) is the main etiology of HCC, it has been reported that Hepatitis B protein x (HBx) plays an important additional role in the promotion of the switch in the gene expression especially in hypoxic tumor microenvironment. The antigen enhances hypoxia signaling through HIF1α activation, enhamces EpCAM expression by activating β-catenin and regulates the EpCAM promoter methylation. [26]

Zhang et al. studied radiomics and demonstrated that lesions with arterial-hyper enhancement and well-circumscribed borders are more susceptible to TACE. Identification of responders may be even more important in patients receiving TACE/TAE as a bridging therapy to transplantation to allow the best use of available organs [27]. Post-embolisation transient hypertransaminesemia can also be an indicator of response to embolisation procedures [28]. Further evaluation of immunological responses of TACE is important and its influence on the higher incidence of metastatic disease following TACE [29,30], as well as the benefits of immunomodulation of TACE with immunotherapy [31], are being reported. Future studies should also consider evaluating other emerging locoregional therapies, such as hepatic arterial infusion chemotherapy (HAIC) [32] and internal radiotherapy.

This study has limitations to note. Firstly, response rate and survival data were affected by moderate/high heterogeneity. Secondly, the low number of included studies requires caution in interpreting the findings. However, the study restricted the inclusion of RCTs only to provide robust and reliable outcome estimates. There are also several newer systemic treatment options and future studies should consider evaluating the benefit of invasive versus non-invasive options and create the tools to identify what will be the best option for the patient based on the radiomics and genomics. Future studies evaluating the impact of embolisation procedures on the liver function (Child–Pugh status) are also needed as a deterioration in the liver function might preclude the sequential and combined immunotherapy/tyrosine kinase inhibitors [32,33].

## 6. Conclusions

In summary, this study provides an update to the existing literature on this topic and broadens the spectrum of considered outcomes. It supports previous analyses showing a non-superiority of TACE over TAE and provides a starting point to consider more work in the future to improve the existing data and find deeper patterns and relationships in this treatment to best deploy its use for a growing global health burden of HCC.

## Figures and Tables

**Table 1:** Summary of search terms used for literature review.

#	Term	Results
**Medline** ^®^		
1	*Carcinoma, Hepatocellular*	17,497
2	*Liver Neoplasms*	25,966
3	HCC	13,454
4	Primary liver cancer	408
5	TACE	1272
6	*Chemoembolisation, therapeutic*	1568
7	Treatment embolization	2
8	Transarterial chemoembolisation	38
9	Transarterial chemoembolization	38
10	TAE	399
11	Transarterial embolisation	16
12	Bland embolisation	1
13	1 OR 2 OR 3 OR 4	27,680
14	5 OR 6 OR 7 OR 8 OR 9 OR 10 OR 10 OR 11	2283
15	12 AND 13	1662
16	Limit 14 to (English language and full text)	1510
**Embase**		
1	*Liver cell carcinoma*	148,941
2	*Liver tumor*	47,443
3	*Hepatocellular carcinoma*	167,582
4	HCC	86,079
5	Primary Liver cancer	3586
6	TACE	10,520
7	*Chemoembolisation*	15,880
8	Transarterial chemoembolisation	401
9	Transarterial chemoembolization	401
10	Treatment embolisation	10
11	TAE	3793
12	Transarterial embolisation	171
13	Bland embolisation	10
14	1 OR 2 OR 3 OR 4	205,638
15	5 OR 6 OR 7 OR 8 OR 9 OR 10 OR 11 OR 12	26,300
16	13 AND 14	17,081
17	Limit 15 to (full text and English language	3867
	**Cochrane Library**	
1	*Carcinoma, Hepatocellular*	5851
2	*Liver Neoplasms*	5958
3	HCC	4158
4	Primary liver cancer	8100
5	TACE	1389
6	*Chemoembolisation, therapeutic*	65
7	Treatment embolization	2063
8	Transarterial chemoembolisation	107
9	Transarterial chemoembolization	708
10	TAE	10,308
11	Transarterial embolisation	242
12	Bland embolisation	35
13	1 OR 2 OR 3 OR 4	16,338
14	5 OR 6 OR 7 OR 8 OR 9 OR 10 OR 10 OR 11	13,543
15	13 AND 14	83,651
16	Limit 14 to (full text and English language)	8710

**Table 2:** Baseline characteristics of included studies.

First Author	Patients, n	Country	Duration	Follow Up (Months)	Chemotherapy Drug	Chemotherapy Dose	Embolisation Agent	Embolisation Agent Size
Brown 2016 [17]	101	USA	2007–2012	48	Doxorubicin	<150 mg	LC bead	100–300, 300–500, 500–700, 700–900 μm
Chang 1994 [18]	46	China	1991–1993	28	Cisplatin	50 mg	Gelatin sponge	1 × 1 mm
Kawai 1992 [19]	289	Japan	1988–1989	36	Adriamycin	40 mg/m^2^	Gelatin sponge	NA
Malagari 2010 [10]	87	Greece	2005–2006	12	Doxorubicin	<150 mg	DC bead	100–300, 300–500 μm
Meyer 2013 [9]	86	UK	2003–2009	36	Cisplatin	50 mg	PVA bead	50–150 μm

**Table 3:** Risk of bias assessment of included randomised controlled trials.

Study Name	Random Sequence Generation	Allocation Concealment	Baseline Differences	Patient Blinding	Carer Blinding	Differing from Intended Treatment	Incomplete Outcome Data	Selective Outcome Reporting	Appropriate Outcome Assessment	Overall
Brown 2016 [17]	Low	Low	Low	Low	High	Low	Low	Low	Low	Low
Chang 1994 [18]	High	High	Low	Unclear	Low	Unclear	Low	Low	Low	Low
Kawai 1992 [19]	High	High	Unclear	Unclear	High	Unclear	Low	High	Low	High
Malagari 2010 [10]	High	High	Low	Unclear	High	Unclear	Low	High	Low	High
Meyer 2013 [9]	Low	High	Low	Unclear	Low	Low	Low	Low	Low	Low

**Table 4:** Summary of meta-analysis of adverse events.

Outcome	OR (95% CI)	*p* Value
Cholecystitis	0.70 (0.12–4.06)	0.7
Liver abscess	0.51 (0.10–2.58)	0.4
Liver failure	1.01 (0.25–4.11)	1.0
Puncture site bleeding	0.96 (0.19–4.93)	1.0
Emesis	11.47 (4.22–31.16)	<0.001
Gastro-intestinal bleed	0.15 (0.01–7.44)	0.3
Leukopenia	1.43 (0.79–2.57)	0.3
Anaemia	2.68 (1.40–5.14)	0.003
Thrombocytopenia	0.89 (0.47–1.68)	0.7
Abdominal Pain	1.12 (0.71–1.78)	0.6
Fever	0.68 (0.41–1.13)	0.1
